# A study to explore the role of a low threshold, fitness focussed physical rehabilitation intervention with protein supplementation to target physical function and frailty in people with problematic substance use and homelessness: protocol for a single-arm feasibility cohort study.

**DOI:** 10.12688/hrbopenres.13678.1

**Published:** 2023-04-25

**Authors:** Fiona Kennedy, Clíona Ní Cheallaigh, Roman Romero-Ortuno, Suzanne Doyle, Julie Broderick

**Affiliations:** 1 Discipline of Physiotherapy, School of Medicine, Trinity College Dublin, Dublin, Dublin, D08WRT, Ireland; 2 Department of General Medicine and Infectious Diseases, St. James's Hospital, Dublin, Dublin, D08W9RT, Ireland; 3 School of Medicine, Trinity College Dublin, Dublin, D08W9RT, Ireland; 4 Discipline of Medical Gerontology, School of Medicine, Trinity College Dublin, Dublin, D08NHY1, Ireland; 5 Mercer's Institute for Successful Ageing, St James Hospital, Dublin, D08NHY1, Ireland; 6 School of Biological and Health Sciences, Technical University of Dublin, Dublin, Ireland

**Keywords:** Inclusion health, addiction, homelessness, exercise, nutritional supplementation

## Abstract

**Background:** People who are homeless are more likely to experience poor mental health and addiction as well as suffering from non-communicable diseases. There is evidence of frailty and accelerated physical ageing among people experiencing homelessness. Appropriate physical rehabilitation and nutritional supplementation strategies can stabilise or reverse frailty and general physical decline, but it is not known how this type of intervention would work in practice in this population.

**Aim:** To evaluate the feasibility and preliminary efficacy of a low threshold physical rehabilitation intervention with protein supplementation to target physical functioning and frailty in people with problematic substance use who are experiencing homelessness.

**Methods:** The intervention will consist of a 12-week low threshold rehabilitation programme with protein supplementation. Participants will be service users of the Ballyfermot Advance Project, a day services centre for people with addiction issues and experiencing homelessness. Primary outcomes will be feasibility including numbers recruited, retention of participants and number of repeat visits. Any adverse events will be recorded. Secondary outcomes will be strength and muscular mass, physical performance and lower extremity physical function, pain, frailty and nutritional status.

**Discussion:** An immediate impact may be simply a distraction from difficult circumstances and potentially an improvement of physical health of participants, which can be a conduit for the emergence of other positive behaviours and recovery. Longer term, this study will generate preliminary data on which to inform the design of a definitive randomised controlled trial of physical rehabilitation and protein supplementation, if indicated.

**Ethics and dissemination:** Ethical approval was granted by the Faculty of Health Sciences Research Ethics Committee in TCD. Study findings will be disseminated through publication into an international peer-reviewed journal and presented at national and international conferences.

## Introduction

Inclusion health is an approach, which aims to prevent and address health and social inequalities of vulnerable people such as those who are homeless ^1^. This is a ‘newer’ area of focus in medicine and the extremely poor health status of people experiencing homelessness is beginning to gain more attention. The collision of disease risk factors with poverty, constant stressors and social exclusion in people experiencing homelessness has demonstrated a markedly elevated rate of non-communicable diseases ^2^. Related to non-communicable diseases and a complex interaction of other factors such as addiction and accidental death, socially excluded populations have a mortality rate that is almost eight times higher than the average for men, and nearly 12 times higher for women ^3^. The median age of death among people experiencing homelessness in Dublin, Ireland is staggeringly low among females at 38 years and 44 years among men ^4^.

Accelerated ageing and earlier geriatric conditions such as falls, poor strength and mobility problems are common in people experiencing homelessness ^5, 6^. A single centre, cross-sectional study, which applied a broad test battery of physical functioning tests to people experiencing homelessness who were admitted for inpatient care, demonstrated that despite a low median age of 45 years, 83% of participants had mobility problems and 70% were frail or pre-frail ^5^.

As frailty is normally a concept associated with ageing ^7^ - the concept of frailty in younger populations can be contentious. Nonetheless, frailty has been identified in younger populations across a number of settings ^8^ and it is recognised that those living in areas of greater deprivation experience the earlier onset of illness and associated disability ^9, 10^. A high prevalence of frailty has been identified in people experiencing homelessness ^5, 11– 15^. Poorer physical health and frailty means people experiencing homelessness have less options for moving to independent housing due to accessibility issues which reinforces the cycle of entrenched homelessness, rough sleeping and dependence on long-term hostel accommodation ^16^. The challenge is to bridge the implementation gap and provide innovative solutions to key challenges faced by people in medium to long term homelessness. While changing the physical status of this cohort will not solve all complex challenges, it is nonetheless a sensible solution focussed target which can be a positive focus from which there can be a ripple effect in terms of outcomes.

Key drivers of physical frailty are poor nutritional intake and sedentary behaviour. Food insecurity is extremely prevalent among people experiencing homelessness ^17^ and may contribute to frailty. It is possible that protein supplementation after exercise may optimise protein synthesis rates ^18^ and help stabilise frailty and physical de-conditioning ^19^. This has been successfully demonstrated in frail older people ^20^. Furthermore, in illicit drug users, exercise can increase the abstinence rate and can reduce withdrawal and anxiety symptoms ^21^.

## Aim and objectives

The overall aim of this study is to test the feasibility and preliminary efficacy of a low threshold physical rehabilitation programme with dietary supplementation to target frailty and poor physical functioning in people who are homeless.

Objectives:

1. To evaluate the adherence (uptake, compliance, number of repeat visits) to the physical rehabilitation and protein supplementation programme.
2. To explore baseline and end of programme physical, nutritional and frailty status, and self-reported pain.
3. To ascertain perceptions of unmet physical health needs, exercise habits and how an exercise intervention should ideally be designed to meet the needs of this cohort with lived experience of homelessness and active addiction issues.

## Methods

### Design and study setting

This single arm feasibility cohort study is taking place in the Ballyfermot Advance Project, located in Dublin 10. The study will commence in October 2022 and will finish in March 2023. The Ballyfermot Advance Project provides a five-day a week meal service, as well as drug and alcohol related services for people with active addiction issues, the majority of whom experience homelessness. A dedicated exercise room in a nearby community centre has been allocated for the duration of the intervention period. This study has received ethical approval from the Faculty of Health Sciences REC at Trinity College Dublin (Ethical Approval Reference Number: 211202.

### Sample selection, recruitment and eligibility screen

A gatekeeper in Ballyfermot Advance has been appointed as the study liaison. The gatekeeper will distribute the Participation Information Leaflet (PIL) and consent form in advance of the study. Staff members with a knowledge of eligible clients who access services in Ballyfermot Advance will inform them of the study and supply them with study related information. Study information leaflets in plain English will be available throughout the centre. Once referred, and the potential participants present to the exercise room, the dedicated research physiotherapist, FK, will do an initial eligibility screen at the point of enrolment to ensure potential participants meet the eligibility criteria.

### Obtaining consent

All potential participants will be provided with a PIL and an exercise information leaflet detailing the purpose of the data collection, the exercise intervention, potential risks and benefits and data protection rights. Due to the expected high levels of functional illiteracy, the research physiotherapist will read the study related information where applicable and will be available to answer any study related queries. Where possible there will be a seven-day gap between receipt of the PIL and obtaining consent to allow potential participations time to consider participation. Due to the anticipated fluctuation in interest levels however, and other competing priorities related to mood, motivation and active addiction issues, flexibility has been built into the consent process. This means that clients who express an interest in the programme and willingness to participate the same day as first receiving the study information can be consented and commence the programme at a time suited to them. This method was successfully employed previously in a cross-sectional study conducted with patients experiencing homelessness in St. James’s Hospital ^5^.

Once the research physiotherapist is satisfied that the potential participant has read (or has been read to) and fully understands the PIL, they will proceed to obtain written informed consent. Obtaining consent will take place at the first interaction with the participant prior to commencement of testing. The written consent informs participants that they are permitted to withdraw from the study at any time. Participants are given their own copy of this consent form and PIL, signed by themselves and the research physiotherapist.

### Inclusion and Exclusion Criteria

The aim of the study is to be as pragmatic and low threshold as possible. This means that minimal constraints are put in place to access the intervention. In order to be as pragmatic as possible in terms of inclusion criteria, all clients (>18 years) accessing services in Ballyfermot Advance who consent to study participation can be included in this study. Only participants with acute problematic behavioural issues or confusion, are in an agitated state or have major physical problems, (medical or orthopaedic) which would preclude ability to safely participate in the exercise class will be excluded from study participation. Participants with a confirmed pregnancy will also be excluded as physical functioning/performance tests scores in advanced stages of pregnancy may vary from baseline values ^22^.

In the design of this study, we were cognisant of the likely complex needs of many participants as complex childhood trauma has been commonly experienced by people who experience homelessness and substance misuse problems. Using a Trauma Informed approach to care ^23^ and based on experience from a previous Inclusion Health undergraduate clinical placement ^24^, the following key pieces of advice were adapted slightly and incorporated in the approach to assessment and follow up with participants; (i) be empathetic and have an open mind, (ii) communication skills are very important, (iii) know when not to intervene (iv) plan in advance use a flexible approach, (v) speak to someone if you feel uncomfortable or unsure about a situation.

### Intervention

The intervention will consist of a twice weekly, 12-week exercise programme with nutritional supplementation. The intervention will be fully supervised and delivered by two research physiotherapists. Group exercise classes or one-to one sessions will be delivered depending on participant preference. Participants will be advised of a schedule of class times, including gender specific classes and will be allocated to a specific class based on their preference. An alternate class will be offered if participants cannot attend at their scheduled time. A ‘Park Walk’ will also be scheduled one day per week. This will be a 30-minute self-paced walk of low intensity led by the research assistant involved in this programme. This is to build up exercise frequency during the week and is building in a habit which it is hoped can be continued by participants beyond the life cycle of the project. Flexibility in programme commencement and completion dates will be provided to enable the 12-week intervention to be completed within a 15-week period of time.

The PAR-Q ^25^, a pre-screening questionnaire, will be conducted on participants prior to commencement of the exercise classes. The research physiotherapist will, with permission, write to the participants General Practitioner (GP) to advise them of their intention to take part in the programme and to clarify that it is safe for them to proceed with the exercise intervention. If the individual does not have a GP, the research physiotherapist will discuss this individual case with a specialist consultant in Inclusion Health based in St. James’s Hospital, Dublin. The case will be outlined in broad terms, without revealing any personal details of the participant, solely as a sounding board as to whether it would be suitable for the participant to attend or not.

The exercise intervention will focus on general fitness and will include resistance, aerobic and functional exercises, with in-built flexibility based on individual participants’ needs. The exercise component will be based on ‘core’ exercises ( Table 1) which will be adjusted to increase or decrease difficulty based on the results of the initial assessment and ability of participants, as judged by the research physiotherapist. Each session will commence with a warm-up and stretch of the major muscles and will end with cool-down and stretch.

**Table 1.** Core Exercises.

Core exercise	Adaptations*
Sit to stand/squats/lunges	use of weights/ball
Elbow Bends	weights
Step-ups	height of step; weights
Arm elevations	weights
‘Penguin waddle’-hip abduction	With additional upper limb abduction and elevation; movement with 360° turns
Scapular retractions	weights
Aerobic activity	ladders, hurdles, skipping ropes, jumping jacks dance, game with cones/balls
Balance	Tandem; single leg stance, upper limb and trunk movements; weights and ball work

Adaptations: exercises individualised and progressed for each participant by research physiotherapist

A low-specification pedometer will be supplied to encourage increasing daily step count and goal setting will be discussed with participants. This is to build a scientifically sound psychological framework into the intervention to encourage motivation to partake in physical activity.

The intensity of the workout will be managed by using the Borg Perceived Rate of Exertion (RPE) scale ^26^ where participants will be advised to exercise at a rate of between 11 and 13 on the PRE scale, i.e. where they find the exercise somewhere between ‘fairly light’ to ‘somewhat hard’, where they find it hard to have a conversation but can comfortably continue to exercise.

To promote post-exercise muscle protein synthesis ^18^, a nutritional supplement (200ml pre-prepared ‘protein shake’ Fresubin, https://www.fresubin.com/) which consists of 20g of protein will be offered to all participants immediately post exercise in the exercise room.

In an attempt to build sustainability beyond the life cycle of the project, participants will also be educated about exercise and available local resources where possible. Participants will be invited and encouraged to return three times weekly to continue with the exercise intervention.

### Adherence

The service provided will be low threshold to facilitate adherence and compliance. The research physiotherapists will make every effort to be flexible and accommodating to participants in terms of their attendances to the exercise classes and the Park Walk. Adherence to the programme will be measured by the uptake, compliance and number of repeat visits to the drop-in programme. Demographic information will include biological sex and current homeless status.

### Demographic details collected

Demographic details, including age, and named GP of participants will be collected. A letter will be sent to each GP to inform them of study participation. Questions around current addiction status will be guided by Section 1 of the Treatment Outcome Profile ^27^. As the research physiotherapist will not have access to participant medical/social records, senior staff of Ballyfermot Advance Project will provide pertinent medical/addiction/social information relating to the participants if required.

## Outcomes

### Primary outcomes

The following feasibility outcomes will be recorded; numbers recruited, retention of participants by number of repeat visits. Any adverse events will also be recorded.

### Secondary outcomes

**1. Strength and muscular mass:** Muscular strength will be estimated ^28, 29^ by using a Digital Hand Dynamometer in a sitting position while the hand is unsupported with the elbow at 90° flexion and the underarm and wrist in neutral. Two measurements will be inputted as part of the SHARE-FI frailty instrument ^30^ and values will also be compared to normative reference values established by Steiber ^31^.

Mid-calf circumference girth will be evaluated as this measure correlates with appendicular muscular mass ^32^. This will be measured using a flexible tape measure at the level of the largest circumference of the calf. Higher scores indicate higher levels of muscular mass. The cut-off value for moderately and severely low calf circumference is 34 cm and 32 cm in males, and 33 cm and 31 cm in females ^33^.

Mid-arm muscle circumference reflects both muscle mass and caloric and protein adequacy, and may be used to signify malnutrition or wasting ^34^. This test has been recommended for use in physical testing of those experiencing homelessness ^35^ due to the high prevalence of lower limb swelling ^36^. The maximum upper arm muscular mass will be measured using a flexible tape measure. Results will be compared to global reference values ^37^.

**2. Physical performance and lower extremity physical function:**

This will be measured using the following physical performance measures:

(i) The 10m Walk Test (10MWT). This test measures walking speed and functional mobility and is recorded in m/s. Gait speed is calculated as total distance/time ^38^.

(ii) The 2minute Walk Test (2MWT). This test of self-paced walking ability and functional capacity assesses a participants’ ability to walk unassisted over a 15m distance, as fast as possible, for two minutes. Rest breaks are permitted and the distance covered is measured ^39^.

(iii) The Chair Stand Test (CST). This test of lower limb strength and endurance measures the total number of sit to stand repetitions a participant can perform in 30 seconds ^40^.

(iv) The Single Leg Stance Test (SLS). This test of balance is performed on each leg. The participant is timed standing unassisted on one leg, with eyes open and hands placed on the hips ^41^.

**3. Pain:** Each participant will be questioned whether they are experiencing any pain and will be questioned about its location and duration. Severity of pain will be assessed using the Numerical Rating Scale (NRS). The NRS is a unidimensional measure of pain intensity from 0-10, with 0 being zero pain and 10 the worst pain imaginable ^42^.

**4. Frailty**: This will be assessed in two ways; using the Clinical Frailty Scale (CFS) ^43^ and the SHARE-FI ^30^. This scale is assessed by the tester and each point on the scale is correlated with a description of frailty along with a visual chart to aid the tester in classifying frailty from 1 (very fit) to 9 (terminally ill). Higher scores indicate higher levels of frailty. The SHARE-FI is a valid tool to measure the level of frailty in individuals aged ≥50 years ^30^. It consists of quick questions related of the following variables; exhaustion, loss of appetite, walking difficulties and low physical activity. Answers are entered into a freely available web calculator to generate a frailty score and a frailty category of non-frail, pre-frail and frail is generated.

**5.** **Nutritional status** will be assessed by using the Mini-nutritional assessment (MNA) score ^44^. The MNA assesses the risk for malnutrition. In particular, the short form of the MNA (MNA-SF) ^45^ is a screening tool consisting of six questions on food intake, weight loss, mobility, psychological stress, or acute disease, the presence of dementia or depression, and body mass index (BMI). The maximum score for this part is equal to 14. A score equal to or higher than 12 indicates that the subject under study has an acceptable nutritional status thus excluding malnutrition and/or malnutrition risk, meanwhile, a score ≤ 11 implicates to proceed with the complete version of the MNA (MNA-LF) ^45^. As this test has not been validated for this population, the terminology of two of the questions of the MNA (regarding acuity of illness and psychological stress) have been slightly modified for the purposes of this study, ie “Have you recently been sick or in hospital?” and “Have you problems with concentration or memory?”

**6. Body Mass Index (BMI).** Height and weight will be measured and the following formula will be applied to generate BMI; kg/m ^2^.

**7. Self-report:**

**Short-Form 12 (SF-12) ^46^.** The SF-12 is a self-report measure of health used across age, disease and treatment groups. It uses eight domains including physical and social activities, pain, mental health, emotional health, vitality and general health perceptions to measure health. The participant completes a 12 question survey which is scored by the researcher. The minimum possible score is 12 and the maximum possible score is 48. Lower scores indicate better health. To ascertain perceptions of unmet physical health needs & rehabilitation/exercise preferences, open-ended questions will be used regarding (i) concerns with current physical health, (ii) exercise history (iii) current concerns/priorities of the participant and (iv) the final questions asks “do you have someone who looks out for you?”. This information will be transcribed by the research physiotherapist and repeated back to the participant to verify accuracy. It will not be audio-recorded.

## Data collection and management

### Analytic plan

Our study is very much feasibility focussed and not hypothesis driven so formal power calculations are not directly applicable. Prospectively, potential participants that meet the study eligibility criteria will be invited to participate. Descriptive statistics will summarise participant demographics and feasibility measures such as attendance rates. Nominal or ordinal variables will be reported as frequencies and percentages. Continuous variables will be summarised as mean and standard deviation if normally distributed and median and inter-quartile range if non-normally distributed. Data will be tested for normality using the Kolmogorov–Smirnov test and will be compared across timepoints using the general linear model procedure (normally distributed data) and the Friedman’s test (non-normally distributed data). As participants will be heterogeneous, data will be sub-stratified and participants will be grouped meaningfully. Free text responses from subjective questions will be reported and organised into topic areas.

## Funding

This has been funded by Trinity College Dublin and the Ballyfermot Advance Project.

## Dissemination plans

Conference presentations and publications in peer-reviewed journals will be one method of dissemination. These will be done following the data analysis.

## Study status

Recruitment and data collection will commence on October 3rd 2022 and will be completed by March 2023.

## Discussion

This protocol describes a novel and pragmatic, low threshold intervention which aims to address the known poor physical health condition of people experiencing homelessness and problematic substance use. Given this is such a novel area there is no comparator group. This study will nevertheless increase knowledge, understanding and awareness of the physical health needs of this population and facilitate a better understanding of unmet need, thus assisting in shaping future physical rehabilitation services to suit these complex and transient needs. It is hoped that this study will provide preliminary data to optimise the intervention and inform the design of a definitive randomised controlled trial, where applicable. An immediate impact may be an improvement in physical health of participants, which can be a conduit for the emergence of other positive behaviours and recovery. Overall, this research will address an intractable global societal challenge, have wide impact and improve the quality of life, health and well-being of some of our most vulnerable citizens.

## Data Availability

Data from this study will be available in open access form. No data are associated with this article.
