# Supplementary material for: A study to explore the role of a low threshold, fitness focussed physical rehabilitation intervention with protein supplementation to target physical function and frailty in people with problematic substance use and homelessness: protocol for a single-arm pre-post intervention study
Source: HRB Open Res. 2024 Oct 29;6:26. Originally published 2023 Apr 25. [Version 2] doi: 10.12688/hrbopenres.13678.2 (PMC11576564; doi:10.12688/hrbopenres.13678.2)
Supplement: Peer review history

# Reviewer report: Reviewer response for version 2

**DOI:** 10.21956/hrbopenres.15384.r42998

**Published:** 2024-11-19

**Authors:** Catriona Connell

**Affiliations:** 1 University of Stirling, Stirling, Scotland, UK

Thank you for the detailed responses to my review. The amended manuscript reads clearly, and I look forward to seeing results. There a couple minor editing issues (outcomes section has a cut and paste error) but otherwise there is sufficient detail here for reviewers of the results to follow any study changes. 

 Well done for sticking with this commitment to open science despite such a long wait to find reviewers.

Is the study design appropriate for the research question?

Yes

Is the rationale for, and objectives of, the study clearly described?

Partly

Are sufficient details of the methods provided to allow replication by others?

Partly

Are the datasets clearly presented in a useable and accessible format?

Not applicable

Reviewer Expertise:

Applied health research in inclusion health (particularly justice, substance use, homelessness, mental health)

I confirm that I have read this submission and believe that I have an appropriate level of expertise to confirm that it is of an acceptable scientific standard.

# Reviewer report: Reviewer response for version 2

**DOI:** 10.21956/hrbopenres.15384.r42997

**Published:** 2024-11-06

**Authors:** JUAN TORTOSA-MARTÍNEZ

**Affiliations:** 1 University of Alicante, Alicante, Spain

The authors have clarified nicely the points raised in my first revision.

Is the study design appropriate for the research question?

Yes

Is the rationale for, and objectives of, the study clearly described?

Yes

Are sufficient details of the methods provided to allow replication by others?

Partly

Are the datasets clearly presented in a useable and accessible format?

Yes

Reviewer Expertise:

Exercise for the health and quality of life of special populations

I confirm that I have read this submission and believe that I have an appropriate level of expertise to confirm that it is of an acceptable scientific standard.

# Reviewer report: Reviewer response for version 1

**DOI:** 10.21956/hrbopenres.14958.r41844

**Published:** 2024-09-02

**Authors:** Catriona Connell

**Affiliations:** 1 University of Stirling, Stirling, Scotland, UK

Thank you for the opportunity to review this protocol for a 'low threshold fitness focused physical rehabilitation intervention with protein supplementation to target physical function and frailty in people with problematic substance use and homelessness'. It is positive to see intervention research by allied health professionals in this field. 

 I note that this protocol was published online and peer reviewed initially over a year ago, and that the dates of the study were such that even at that point the study should have concluded. As such providing comments now will be of no utility for designing the study, but I hope may assist in reporting the results.

 Results of a similar study were published as a conference abstract in 2022, although it appears the intervention may have been developed. This prior study should be referenced and the adaptations to the intervention made clear. 

 A note on language. The authors use different terminology for substance use, including problematic substance use, illicit drug users, and addiction. It would be useful to select one term or make clear if these refer to different concepts. In some areas the term 'people who use drugs/alcohol' is preferred, or people with problematic use of drugs/alcohol/substances. 

**Abstract:** 

 The aim of the study is to establish feasibility and preliminary efficacy, both need to be clearly defined given the use of the term efficacy tends to be associated with more robust designs (e.g., controlled study with comparator group). 

**Introduction:** 

 First sentence has an unnecessary comma after approach. Can you support the argument that homelessness/inclusion health is attracting more attention (in what sense, research, practice, policy, all?). Would also be useful to outline what homelessness is (i.e. are you considering rooflessness, or people living in temporary/unsuitable accommodation, sofa-surfing etc).

 Paragraph 2-3 discuss pre-frailty and frailty. It would be useful to explain what these conditions are/look like for non-expert readers.

 Paragraph 3 - Is there evidence to support the claim that physical disability this limits accommodation options and reinforces severe and multiple disadvantage (this would seem obvious but would be good to support this more clearly). This should be fewer options rather than less. Authors describe the challenge as being the implementation gap, but there isn’t a clear account and evidence that there are a range of effective interventions not being implemented. Please define ‘medium- to long-term homelessness’. Final sentence is a bit long. Consider shorter clearer sentences.

 Paragraph 4 – reference needed for statement that frailty is driven by poor nutrition.

**Aims and objectives**

 As above re term ‘preliminary efficacy’.

 In objectives the term adherence is used for the first time. This is slightly different to feasibility as stated in your aim and abstract. Consistency of language needed here and in what you are considering. Objective 2 could be more explicit – to examine change in measures of … Objective 3, examining perceptions of unmet need, is not clearly reflected in the aims and abstract and needs a rationale. I agree with reviewer one that it would be (have been) useful to include qualitative process-type evaluation approaches to collecting and analysing this data, taking perceptions of participants, service providers and funders.

**Methods**

 This is described as a single arm feasibility cohort study. ‘Cohort’ is not the correct terminology for your design, which is used in longitudinal observational studies rather than intervention studies like yours. Feasibility study is adequate.  

 As a feasibility study, you need to be explicit in advance in what would be the determining factor of feasibility. Is it that the intervention is feasible to deliver in practice, or is it that you need to know whether an RCT is feasible, or both. E.g., do you need to be able to recruit XX over a certain period of time, what proportion of drop out can you accommodate, what is considered sufficient adherence to gain a dose of the intervention and thus how much do people need to come etc…   Such specificity may be challenging at your stage of intervention development but it is still good practice to define what results you consider would indicate feasibility. Obviously this is no use now the study is concluded, but you can consider these in your main manuscript in the discussion of whether a full RCT is feasible.

 You are very explicit about the study site (I am not sure what Dublin 10 is?) and should be careful that this does not risk identifying participants, this depends largely on how many people may have been using the service during the study. However, you have ethical approval so assume you have justified this.

 Consenting process looks appropriate. At this point recruitment, consenting and I assume measures are all done by the person providing the intervention (I think, although who conducts the measures is unclear) and therefore it would be useful to note the limitations of this in introducing bias.  Inclusion and exclusion, how will you ascertain ‘acute problematic behavioural issues’’ and what does this mean. Given you are working with people who use alcohol/drugs,  it will be vital that there is ongoing attention to capacity. This is vital at the consenting stage but doesn’t mean someone needs to be completely excluded. You mention trauma informed care and your adaptation of these. I am not sure who these are for and what ‘communication skills are very important’ means. It would be useful to expand on how this related to physical rehabilitation, particularly where there may be physical contact with participants.

**Intervention**

 You need to have a much clearer articulation of the intervention components, and the hypothesised mechanisms of change. Using the TiDIER checklist ( https://www.bmj.com/content/348/bmj.g1687) and presenting an intervention logic model will be helpful. It is a little unclear at present what each persons ‘dose’ would be. Is it one group and one park walk? Additional steps and goals? Three times weekly?

 The PARQ- should be conducted with rather than on participants? In contacting the GP this is a good idea for obtaining information about contraindications, however, what if the person does not consent/a response is not forthcoming. This may be something that needs to be adapted to optimise feasibility.

 Adherence (see above comment) – uptake, is that people recruited who actually engage, or the proportion of eligible day service clients who take up the intervention? What indicated compliance. Demographics included here but should be all together.

**Outcomes**

 Retention – useful to track dop out and point of drop out. Would also be vital to know how much prompting and support is required to facilitate attendance at the off site location.

 I can’t comment in detail on the physical outcome measures but just note that will need to add details of any known reliability and validity data where appropriate  

 IN terms of unmet need and wider input on feasibility, barriers and facilitators and intervention revisions and planning, a qualitative approach with a wider participant base would be helpful.  

**Data collection and management**

 No detail of who is collecting the data at what points. Analytical plan is light and could be more robust in explaining which tests will be used for each outcome depending on the type of data it produces. As there is no detail of the anticipated sample size, or of how the GLM will be constructed this is difficult to comment on. GLM is the first mention of timepoints, is there an interim measure or only pre-post?  

**Dissemination**

 It may be beneficial to consider dissemination beyond academic audiences, particularly to gain input from practitioners, policymakers and people experiencing homelessness and problematic substance use on issues relating to intervention optimisation and delivery in 'real world' practice. 

**Additional recommendations**

 When reporting your study, you should use CONSORT- extension for feasibility and pilot studies and include this as a supplementary file. See the EQUATOR (Enhancing the QUAlity and Transparency Of health Research) website ( www.equator-network.org/), and this article for some additional guidance [1].

Is the study design appropriate for the research question?

Yes

Is the rationale for, and objectives of, the study clearly described?

Partly

Are sufficient details of the methods provided to allow replication by others?

Partly

Are the datasets clearly presented in a useable and accessible format?

Not applicable

Reviewer Expertise:

Applied health research in inclusion health (particularly justice, substance use, homelessness, mental health)

I confirm that I have read this submission and believe that I have an appropriate level of expertise to confirm that it is of an acceptable scientific standard, however I have significant reservations, as outlined above.

## References

[1] : Guidelines for reporting non-randomised pilot and feasibility studies. *Pilot and Feasibility Studies* .2019;5(1) : 10.1186/s40814-019-0499-1 10.1186/s40814-019-0499-1 31608150 PMC6778655

# Response

**Published:** 2024-09-26

**Authors:** Fiona Kennedy

**Affiliations:** Trinity University, Ireland

Dear Dr. Connell,

 Thank you very much for your feedback and the opportunity to revise this manuscript. Please see responses to each point raised. We feel the review process has significantly improved the quality of this protocol and very much hope this now meets the criteria for approval.

 Best Wishes,

 Fiona Kennedy and co-authors

**Comment 1: **Results of a similar study were published as a conference abstract in 2022, although it appears the intervention may have been developed. This prior study should be referenced and the adaptations to the intervention made clear. 

**Response:** Thank you for this point. I believe this conference abstract you are referring to related to this and a previous linked study which was presented just after this protocol was submitted. It has been since published as a full paper:

 (the LEAP I trial, doi: 10.1371/journal.pone.0301926).

**Comment 2**: A note on language. The authors use different terminology for substance use, including problematic substance use, illicit drug users, and addiction. It would be useful to select one term or make clear if these refer to different concepts. In some areas the term 'people who use drugs/alcohol' is preferred, or people with problematic use of drugs/alcohol/substances. 

**Response:** **Thank you. This has been amended and terminology is consistent throughout.**

**Amendment:** **‘A study to explore the role of a low threshold, fitness focussed physical rehabilitation intervention with protein supplementation to target physical function and frailty in people who experience homelessness and addiction’** **.**

**Abstract:** 

**Comment 3**: The aim of the study is to establish feasibility and preliminary efficacy, both need to be clearly defined given the use of the term efficacy tends to be associated with more robust designs (e.g., controlled study with comparator group). 

**Response:** This has been amended to establish feasibility and ‘pre-post intervention impact’, as per further discussion with study collaborators since submitting the protocol and has been used for reporting of the results. This is reflected in the title, in comment 2 above and is also explained in the abstract and main article.

**Amendment:** **‘the aim of this study is to test the feasibility and pre-post intervention impact’.**

**Introduction:** 

**Comment 4.**  First sentence has an unnecessary comma after approach.

**Response:** **Amended, thank you.**

**Comment 5.** Can you support the argument that homelessness/inclusion health is attracting more attention (in what sense, research, practice, policy, all?).

**Response:** **Upon reflection it was felt that this sentence lacked specificity and has since been removed.**

**Comment 6:** Would also be useful to outline what homelessness is (i.e. are you considering rooflessness, or people living in temporary/unsuitable accommodation, sofa-surfing etc). **Response:** **Yes, the European Typology of Homelessness and Housing definition was used.** **European Typology of Homelessness and Housing Exclusion https://www.feantsa.org/download/en-16822651433655843804.pdf**

**(line 57, reference 4)**

**Comment 7:** Paragraph 2-3 discuss pre-frailty and frailty. It would be useful to explain what these 5. conditions are/look like for non-expert readers.

**Response**: **Frailty and pre-frailty are now defined.**

**Amendment:** **Frailty ‘a complex multidimensional state of physiological vulnerability’** **and pre-frailty its ‘prodromal stage’.**

**Comment 8:** Paragraph 3 - Is there evidence to support the claim that physical disability limits accommodation options and reinforces severe and multiple disadvantage (this would seem obvious but would be good to support this more clearly). This should be fewer options rather than less. 

**Response:** **Have referenced a report which outlines the difficulties of exiting homelessness when living with a disability (reference 21:** **Homelessness and disability in the UK Report. Centre for Homelessness Impact. 2023.** **Accessed October 2024.** **https://cdn.prod.website-files.com/59f07e67422cdf0001904c14/645a76da097c6dad33fcc423_CHI-disabilities-homelessness23.pdf** **).** **Amended to ‘fewer options’.**

**Comment 9.** Authors describe the challenge as being the implementation gap, but there isn’t a clear account and evidence that there are a range of effective interventions not being implemented.

**Response:** **The authors are referring to the need to implement new or ‘innovative’ strategies (in addition to existing interventions) to target physical disability/function as a way to help people who are faced with the aforementioned challenges.**

**Comment 10.** Please define ‘medium- to long-term homelessness’.

**Response:** **Long term homelessness is defined by many as chronic homelessness or those who have been in sheltered accommodation for a prolonged period. For the purpose of this section, this has been amended to long term homelessness.**

**Comment 11**: Final sentence is a bit long. Consider shorter clearer sentences.

**Response:** **Amended.**

**Amendment:** ‘ **Improvements in physical health will not solve all of the complex challenges faced by this population, it is nonetheless a sensible and positive solution focussed target which may have a ripple effect’.**

**Comment 12: ** Paragraph 4 – reference needed for statement that frailty is driven by poor nutrition.

**Response:** **Amended.**

**Amendment:** **Reference no 22 :** **Pérez-Zepeda MU, Castrejón-Pérez RC, Wynne-Bannister E, García-Peña C. Frailty and food insecurity in older adults. Public Health Nutr. 2016;19(15):2844-9.**

**Aims and objectives**

**Comment 13: **As above re term ‘preliminary efficacy’.

**Response:** **Amended to ‘pre-post intervention impact’.**

**Comment 14:** In objectives the term adherence is used for the first time. This is slightly different to feasibility as stated in your aim and abstract. Consistency of language needed here and in what you are considering.

**Response:** **Apologies, adherence was used in the objectives to breakdown how feasibility was measured (ie. recruitment, retention and adherence). For improved clarity, this has been amended.**

**Amendment: ‘** **To evaluate recruitment, retention and adherence to the physical rehabilitation and protein supplementation programme’.**

**Comment 15:** Objective 2 could be more explicit – to examine change in measures of …

**Response:** **Amended**

**Comment 16**: Objective 3, examining perceptions of unmet need, is not clearly reflected in the aims and abstract and needs a rationale. I agree with reviewer one that it would be (have been) useful to include qualitative process-type evaluation approaches to collecting and analysing this data, taking perceptions of participants, service providers and funders.

**Response: Thank you for this comment. Since the protocol was written, a further amendment was made to ethics to include exit surveys, which we did. This is also part of the reason we amended the title of the study to ‘impact’ rather than efficacy/effectiveness.**

**Methods**

**Comment 17**: This is described as a single arm feasibility cohort study. ‘Cohort’ is not the correct terminology for your design, which is used in longitudinal observational studies rather than intervention studies like yours. Feasibility study is adequate.  

**Response:** **Thank you for this comment. Amended.**

**Comment 18:** As a feasibility study, you need to be explicit in advance in what would be the determining factor of feasibility. Is it that the intervention is feasible to deliver in practice, or is it that you need to know whether an RCT is feasible, or both. E.g., do you need to be able to recruit XX over a certain period of time, what proportion of drop out can you accommodate, what is considered sufficient adherence to gain a dose of the intervention and thus how much do people need to come etc…   Such specificity may be challenging at your stage of intervention development but it is still good practice to define what results you consider would indicate feasibility. Obviously, this is no use now the study is concluded, but you can consider these in your main manuscript in the discussion of whether a full RCT is feasible.

**Response:** **Thank you for this valuable point. As this type of study is so novel, it did not specifically define what results would define feasibility, rather it evaluated the feasibility of how this intervention would work in practice, therefore explored recruitment, retention and adherence to the programme, as stated in the aims and objectives.**

**Comment 19: **You are very explicit about the study site (I am not sure what Dublin 10 is?) and should be careful that this does not risk identifying participants, this depends largely on how many people may have been using the service during the study. However, you have ethical approval so assume you have justified this.

**Response:** **Amended this description.**

**Amendment:** **‘taking place in a suburban area of Dublin with high levels of deprivation’.**

**Comment 20**: Consenting process looks appropriate. At this point recruitment, consenting and I assume measures are all done by the person providing the intervention (I think, although who conducts the measures is unclear) and therefore it would be useful to note the limitations of this in introducing bias. 

**Response:** **Yes recruitment, consent and measures were conducted by the person providing the intervention but also with a research physiotherapist and often a member of staff from the centre present. This has now been acknowledged this as a limitation in the discussion, thank you.**

**Amendment:** **‘** **A further limitation of this study is the same personnel conducting both evaluation and the intervention, which may introduce bias’.**

**Comment 21:** Inclusion and exclusion, how will you ascertain ‘acute problematic behavioural issues’’ and what does this mean. Given you are working with people who use alcohol/drugs, it will be vital that there is ongoing attention to capacity. This is vital at the consenting stage but doesn’t mean someone needs to be completely excluded.

**Response:** **This comment intended to highlight that if a person’s behaviour was problematic (ie. putting themselves, class participants or the researcher at risk), they would then be excluded. We used the term ‘acute problematic behavioural issues’ as it was considered a dynamic process and needed to be assessed at the initial consent stage and also at each interaction given the potential fluctuating presentation of participants. This has been amended.**

**Amendment:** **‘P** **articipants with problematic behavioural issues, including confusion or extreme agitation or who have major physical problems (medical or orthopaedic), which would preclude ability to safely participate  in the exercise class will be excluded from study participation. Assessment of behaviour will be a dynamic process and conducted at each interaction due to possible fluctuating presentations** **’**

**Comment 22:** You mention trauma informed care and your adaptation of these. I am not sure who these are for and what ‘communication skills are very important’ means. It would be useful to expand on how this related to physical rehabilitation, particularly where there may be physical contact with participants.

**Response:** **Thank you for this point. This section has been amended.**

**Amendment:** ‘ **Using a Trauma Informed approach to care ^29^ and based on experience from a previous Inclusion Health undergraduate clinical placement ^30^, the following were incorporated in the approach to assessment and follow up with participants; (i) empathy, (ii) consistency (iii) understanding and (iv) flexibility’.**

**Intervention**

**Comment 23:** You need to have a much clearer articulation of the intervention components, and the hypothesised mechanisms of change. Using the TiDIER checklist ( https://www.bmj.com/content/348/bmj.g1687) and presenting an intervention logic model will be helpful.

**Response:** **Thank you. The Tidier checklist has since been used for the full study write-up.**

**Comment 24:** It is a little unclear at present what each persons ‘dose’ would be. Is it one group and one park walk? Additional steps and goals? Three times weekly?

**Response:** **Apologies for the lack of clarity. Amended.**

**Amendment: ‘** **The intervention will consist of three exercise opportunities, including a twice weekly, 12-week exercise class with nutritional supplementation’ and A ‘Park Walk’ will also be scheduled one day per week’.**

**Comment 25.** The PAR-Q-should be conducted with rather than on participants?

**Response:** **Amended**

**Comment 26.** In contacting the GP this is a good idea for obtaining information about contraindications, however, what if the person does not consent/a response is not forthcoming. This may be something that needs to be adapted to optimise feasibility. **Response:** **Thank you for this point. Considering this study involved an exercise intervention in a population who experience chronic medical conditions, and who also exercise minimally and have exercise- risk factors, (identified by the PAR-Q), it was deemed necessary to get medical consent from a medical practitioner. This was discussed and agreed with one of the study collaborators, a consultant in Inclusion Health. This was outlined in the consent form.**

**Comment 27.** Adherence (see above comment) – uptake, is that people recruited who actually engage, or the proportion of eligible day service clients who take up the intervention?

**Response:** **Apologies for the lack of clarity. Adherence refers to those who adhered to the exercise programme and the protein supplement, consistent with a preceding study **(the LEAP I trial, doi: 10.1371/journal.pone.0301926). **and this line has been amended.**

**What indicated compliance. ‘Compliance’ removed to avoid confusion. The section on primary outcomes has been amended.**

**Amendment**: ‘ **The following feasibility outcomes will be recorded; numbers recruited, retention of participants including number of  repeat visits and adherence. Any adverse events will also be recorded.**

**Recruitment and retention:** **The numbers recruited and frequency of attendances will be recorded. Participants will be encouraged to attend all sessions if possible. Drop out will also be recorded.**

**Adherence:** **The research physiotherapists will make every effort to be flexible and accommodating to participants in terms of their attendances and adherence to the programme. Adherence will be measured by the adherence to the exercise programme and the protein supplement.**

**Outcomes**

**Comment 28: **Retention – useful to track drop out and point of drop out. Would also be vital to know how much prompting and support is required to facilitate attendance at the off-site location.

**Response:** **Thank you for this point. This was intended and was included in the write-up when this study was completed and has been brought forward to a follow-up study.**

**Amendment:** **‘** **Recruitment and retention: The numbers recruited, and the frequency of attendances will be recorded. Participants will be encouraged to attend all sessions if possible. Drop out will also be recorded’.**

**Comment 29.** I can’t comment in detail on the physical outcome measures but just note that will need to add details of any known reliability and validity data where appropriate.

**Response: A supplementary figure will be included to provide detail of validity and reliability of outcome measures.**

**Comment 30**: In terms of unmet need and wider input on feasibility, barriers and facilitators and intervention revisions and planning, a qualitative approach with a wider participant base would be helpful.

**Response:** **Thank you for this point and as per point above, a subsequent ethical amendment was made, related to permission to add a qualitative element to this study which has taken place and will be reported in the results.**

**Data collection and management**

**Comment 31**: No detail of who is collecting the data at what points. Analytical plan is light and could be more robust in explaining which tests will be used for each outcome depending on the type of data it produces.

**Response:** **More detail has been added to the analytical plan (see point 31).**

**Amendment:** **“Data will be collected pre and post intervention for those who complete the programme, by the research physiotherapist”.**

**Comment 32:** As there is no detail of the anticipated sample size, or of how the GLM will be constructed this is difficult to comment on. GLM is the first mention of timepoints, is there an interim measure or only pre-post?

**Response:** **This has now been amended to reflect the pre-post intervention design of this study.**

**Amendment: ‘** **Results will be compared to evaluate change over time from initial to final intervention. Normally distributed data will be compared from initial to final recorded time-points using paired t-tests and non-normally distributed data via the Wilcoxin-sign rank test’.**

**Dissemination**

**Comment 33:** It may be beneficial to consider dissemination beyond academic audiences, particularly to gain input from practitioners, policymakers and people experiencing homelessness and problematic substance use on issues relating to intervention optimisation and delivery in 'real world' practice.

**Response:** **Thank you for this important point and this has been done since the study completed.**

**Amendment:** **‘** **Results will also be presented to the key stakeholders including people with lived experience and also the funders of this study’** **.**

**Additional recommendations**

**Comment 34:** When reporting your study, you should use CONSORT- extension for feasibility and pilot studies and include this as a supplementary file. See the EQUATOR (Enhancing the QUAlity and Transparency Of health Research) website ( www.equator-network.org/), and this article for some additional guidance [1].

**Response: Thank you for this advice which we will take on board.**

# Reviewer report: Reviewer response for version 1

**DOI:** 10.21956/hrbopenres.14958.r34949

**Published:** 2023-07-18

**Authors:** JUAN TORTOSA-MARTÍNEZ

**Affiliations:** 1 University of Alicante, Alicante, Spain

The present manuscript is a protocol for a feasibility study about a physical rehabilitation program with protein supplementation in people with problematic substance use and homelessness. The topic of health inclusion is very relevant and deserves more intervention programs and further research. The potential value of this program for the quality of life of this population should be recognized.

 The manuscript is well drafted but it requires some minor changes. The introduction section flows well although there should be information about previous exercise and nutrition programs for this population. This would help to understand better the choices made in the design of the program and identify the gaps in the literature. This would be the main required change.

 In the methods section, it is stated that “The study will commence in October 2022 and will finish in March 2023.” This means that the protocol is being reviewed after the study has finished. This is unfortunate, as any comments or suggestions made by reviewers will not be considered for the intervention itself. It is also strange to see that it has been published in April 2023 and read that the study “will” commence in October 2022. However, this can´t be changed now.

 The exercise program includes most information regarding the FIIT principles. The length, frequency and intensity of the program are reported but the duration of the sessions (other than the park walks) are not. Although it is stated that the exercises “will be adjusted to increase or decrease difficulty based on the results of the initial assessment and ability of participants, as judged by the research physiotherapist”, the sessions need more detail about the initial number of repetitions and sets of the strength and balance exercises, rest periods, and the specific duration of the aerobic exercises. In this regard, it would be advisable to plan a progression in the volume and/or intensity. 

 In the sessions, I am not sure if the exercises will be performed in the order presented in Table 1 or if it is just a list of exercises included. The order is important when mixing aerobic, strength and balance exercises in the same session and should be clarified. If the order is as presented in the table, I don´t think performing balance training the last is the best option. I would also include at least one exercise that targets the core muscles although perhaps there is some core muscle training in the “upper limb and trunk movements; weights and ball work” but I can´t tell with the limited description available. 

 Please add the aforementioned details about the exercise program so it can be replicated. 

 The primary outcomes are numbers recruited, retention of participants by number of repeat visits and adverse events. I think this information could be complemented with qualitative data gathered from the points of views of all participants in the program (eg. interviews), the ones that completed the program but also the ones that dropped the program. Understanding the reasons why people dropped the program or why they stayed (adherence) is of special interest to analyse feasibility and improve the design of future programs. This information could be triangulated with the opinions of the physiotherapists (e.g. research diary) and perhaps from staff members of the Ballyfermot Advance (e.g. interviews). It would certainly help to achieve objective 3 of the study “To ascertain perceptions of unmet physical health needs, exercise habits and how an exercise intervention should ideally be designed to meet the needs of this cohort with lived experience of homelessness and active addiction issues.”

 The secondary outcomes are strength and muscular mass; physical performance and lower extremity physical function; pain; frailty; nutritional status; BMI; and the SF-12. I would have included the Timed Up and Go Test as it is one of the most widely used tests for physical performance and is also a measure of dynamic balance (the included Single Leg Stance measures static balance). Again, these secondary outcomes would complement nicely with a qualitative perspective, especially about the perceived benefits of the program, and would contribute to objective 3 of the study. As stated in the abstract, “an immediate impact may be simply a distraction from difficult circumstances”, or there is an increase is self-esteem, or perhaps some other unexpected psychological or social benefits. Without asking participants about their perception, these types of benefits may be ignored when they can be of great relevance for the quality of life of a population with mental health problems and social exclusion. The information could be triangulated just the same way as with the main outcome. However, as it seems as the study already took place it may not be changed now but could be considered for future studies.

Is the study design appropriate for the research question?

Yes

Is the rationale for, and objectives of, the study clearly described?

Yes

Are sufficient details of the methods provided to allow replication by others?

Partly

Are the datasets clearly presented in a useable and accessible format?

Yes

Reviewer Expertise:

Exercise for the health and quality of life of special populations

I confirm that I have read this submission and believe that I have an appropriate level of expertise to confirm that it is of an acceptable scientific standard, however I have significant reservations, as outlined above.

# Response

**Published:** 2024-09-26

**Authors:** Fiona Kennedy

**Affiliations:** Trinity University, Ireland

Dear Dr. Tortosa-Martínez,

 Many thanks for your feedback and the opportunity to revise this manuscript. Apologies for the delay while awaiting a second review. Please see responses to each point raised. We very much hope the revised manuscript now meets the criteria for approval.

 Best Wishes,

 Fiona Kennedy and co-authors

**Comment 1**: The manuscript is well drafted, but it requires some minor changes. The introduction section flows well although there should be information about previous exercise and nutrition programs for this population. This would help to understand better the choices made in the design of the program and identify the gaps in the literature. This would be the main required change.

**Response**: **Thank you for this comment. Amendment will be made to reflect this gap in the literature. Most of the literature on physical activity/exercise interventions targeting this populations are multi-modal interventions and do not specifically focus on exercise and nutrition. To our knowledge, this is the first study, based on a pilot study, which provided an exercise and nutritional intervention in this population.**

**Amendment**: **‘** **There is a dearth of research exploring physical activity and nutritional interventions in this population.** **Kendzor et al, 2017 investigated the effects of a diet and exercise intervention in homeless adults in a randomised controlled trial. This study, however, did not provide a structured, supervised exercise programme. The intervention involved the provision of educational newsletters, healthy snacks, and pedometers with advice on physical activity.** **T** **his study is the first of its kind which will provide a structured exercise and nutritional intervention in this population’.**

**Comment 2**: In the methods section, it is stated that “The study will commence in October 2022 and will finish in March 2023.” This means that the protocol is being reviewed after the study has finished. This is unfortunate, as any comments or suggestions made by reviewers will not be considered for the intervention itself. It is also strange to see that it has been published in April 2023 and read that the study “will” commence in October 2022. However, this can´t be changed now.

**Response**: **Thank you for your comment. This study was submitted for publication in advance of completion of the programme, however, due to the difficulty of initially getting reviewers, inevitable and protracted delays occurred. We feel revising the protocol is still a worthwhile endeavour to increase its quality.**

**Comment 3**: The exercise program includes most information regarding the FITT principles. The length, frequency and intensity of the program are reported but the duration of the sessions (other than the park walks) are not. Although it is stated that the exercises “will be adjusted to increase or decrease difficulty based on the results of the initial assessment and ability of participants, as judged by the research physiotherapist”, the sessions need more detail about the initial number of repetitions and sets of the strength and balance exercises, rest periods, and the specific duration of the aerobic exercises. In this regard, it would be advisable to plan a progression in the volume and/or intensity. 

**Response:** **Apologies for this omission in the programme design. The target duration of the exercise circuit was approximately 20 minutes, based on learning from a pilot study in a similar cohort. For the initial session the target for each exercise was 2 sets of 10-15 repetitions with a rest period between sets, building to 3 sets of 15 repetitions and following this using weights or resistance bands for progression of strength. The aerobic activity was timed and commenced with a minimum of 2 minutes and progressed weekly. More detail is provided in Table 1 in the manuscript.**

**Amendment**: **‘** **Each session, initially of approximate 20 minutes duration, will commence with a warm-up and stretch of the major muscles and will end with cool-down and stretch’**

**Comment 4**: In the sessions, I am not sure if the exercises will be performed in the order presented in Table 1 or if it is just a list of exercises included. The order is important when mixing aerobic, strength and balance exercises in the same session and should be clarified. If the order is as presented in the table, I don´t think performing balance training the last is the best option. I would also include at least one exercise that targets the core muscles although perhaps there is some core muscle training in the “upper limb and trunk movements; weights and ball work” but I can´t tell with the limited description available. Please add the aforementioned details about the exercise program so it can be replicated. 

**Response: Apologies for this lack of clarity. The exercise intervention was a circuit class. Each participant started with a different exercise. Upper limb and lower limb exercises were not performed consecutively to facilitate rest and recovery for muscle groups. Aerobic, balance and core exercises were part of this circuit and dependent on where the participant commenced.**

**Amendment: see Table 1 (manuscript).**

**Comment 5**: The primary outcomes are numbers recruited, retention of participants by number of repeat visits and adverse events. I think this information could be complemented with qualitative data gathered from the points of views of all participants in the program (eg. interviews), the ones that completed the program but also the ones that dropped the program. Understanding the reasons why people dropped the program or why they stayed (adherence) is of special interest to analyse feasibility and improve the design of future programs. This information could be triangulated with the opinions of the physiotherapists (e.g. research diary) and perhaps from staff members of the Ballyfermot Advance (e.g. interviews). It would certainly help to achieve objective 3 of the study “To ascertain perceptions of unmet physical health needs, exercise habits and how an exercise intervention should ideally be designed to meet the needs of this cohort with lived experience of homelessness and active addiction issues.”

**Response:** **Thank you for this important point. This idea for a more in-depth qualitative analysis to ascertain the perspective of participants regarding dropping out/adhering to the programme emerged early in the programme and an ethics amendment was approved to conduct an exit survey upon completion of the programme to get perspectives from participants who attended and those who did not return. This will be reflected in the results paper.**

**Comment 6:** The secondary outcomes are strength and muscular mass; physical performance and lower extremity physical function; pain; frailty; nutritional status; BMI; and the SF-12. I would have included the Timed Up and Go Test as it is one of the most widely used tests for physical performance and is also a measure of dynamic balance (the included Single Leg Stance measures static balance). Again, these secondary outcomes would complement nicely with a qualitative perspective, especially about the perceived benefits of the program, and would contribute to objective 3 of the study. As stated in the abstract, “an immediate impact may be simply a distraction from difficult circumstances”, or there is an increase is self-esteem, or perhaps some other unexpected psychological or social benefits. Without asking participants about their perception, these types of benefits may be ignored when they can be of great relevance for the quality of life of a population with mental health problems and social exclusion. The information could be triangulated just the same way as with the main outcome. However, as it seems as the study already took place it may not be changed now but could be considered for future studies.

**Response**: **Thank you for your insightful comment. In a previous pilot study, the Short Physical Performance Battery (which includes a chair stand test, a short gait speed test, similar to the TUG, and a balance assessment) was utilised and a ceiling effect was observed in participants who had higher physical functioning. For this reason, we amended the test battery to include a more challenging assessment of balance (SLS), lower limb strength (30 second Chair Stand Test) and gait (10m Walk Test and 2 Min Walk Test).**
